# Randomized multicenter follow-up trial on the effect of radiotherapy on painful heel spur (plantar fasciitis) comparing two fractionation schedules with uniform total dose: first results after three months’ follow-up

**DOI:** 10.1186/s13014-015-0471-z

**Published:** 2015-08-19

**Authors:** Marcus Niewald, Henrik Holtmann, Benjamin Prokein, Matthias G. Hautmann, Hans-Peter Rösler, Stefan Graeber, Yvonne Dzierma, Christian Ruebe, Jochen Fleckenstein

**Affiliations:** Department of Radiotherapy and Radiooncology, Saarland University Hospital, Kirrberger Str. 1, D-66421 Homburg, Germany; Department of Oral and Maxillofacial Surgery, University Hospital of Duesseldorf, Moorenstr. 5, D-40225 Duesseldorf, Germany; Department of Radiotherapy, University Hospital of Regensburg, Franz-Josef-Strauß-Allee 1, D.93053 Regensburg, Germany; Department of Radiooncology and Radiotherapy, University Hospital of Mainz, Langenbeckstr. 1, D.55131 Mainz, Germany; Institute of Medical Biometrics, Epidemiology and Medical Informatics, Saarland University Hospital, Kirrberger Str. 1, D-66421 Homburg, Germany

**Keywords:** Heel spur, Plantar fasciitis, Radiotherapy, Pain relief, Quality of life, Fractionation, Single dose

## Abstract

**Background:**

Our first trial on radiotherapy for painful heel spur published in 2012 comparing the analgesic effect of a standard dose (6 × 1.0Gy within three weeks) to that of a very low one (6 × 0.1Gy within three weeks) resulted in a highly significant superiority of the standard dose arm. In the meantime, experimental data have shown that lower single doses in the range of 0.5 – 0.7Gy might be even more effective than the current standard dose of 1.0 Gy. Therefore, we conducted a second trial comparing the analgesic effect of standard single doses of 1.0Gy to that of low single doses of 0.5Gy using uniform total doses of 6Gy.

**Patients and methods:**

One hundred twenty-seven patients were randomized to receive radiation therapy either with a total dose of 6.0Gy applied in 6 fractions of 1.0Gy twice weekly (standard dose) or with the same total dose applied in 12 fractions of 0.5Gy three times weekly (experimental dose). In all patients lateral opposing 6MV photon beams were used. The results were measured using Visual analogue scale (VAS), Calcaneodynia score (CS) and SF-12 health survey. The first phase of this trial ended after a three months’ follow-up; it will be continued up to 48 weeks.

**Results:**

Nine patients had to be excluded after randomization either due to the withdrawal of informed consent to radiotherapy by the patients or radiotherapy with an incorrect dosage. The groups were comparable concerning biographical and disease data. The mean calcaneodynia score (CS) was higher in the experimental group (*p* = 0.002). After three months’ follow-up, we saw a very favorable pain relief in both arms (decline of VAS score: standard arm 42 points, experimental arm 44 points (n.s.), but we did not notice any statistically significant difference between the arms neither concerning the pain parameters nor the quality of life parameters. No relevant acute side effects were recorded.

**Conclusions:**

Favorable laboratory results could not be translated into an enhanced pain relief in our patients. This trial was terminated after the interim analysis (127 patients randomized). Further trials will be necessary to explore the best fractionation schedule.

This trial has been approved by the expert panel of the DEGRO as well as by the Ethics committee of the Saarland Physicians’ chamber.

**Trial registration:**

Current trial registration at German Clinical Trials Register with the number DRKS00004458

## Background

The painful heel spur or plantar fasciitis was first described by Plettner [1] summarizing radiological findings of exostoses situated at the plantar part of the calcaneus or at the insertion point of the plantar aponeurosis. Various authors give values for the incidence of 8-88 % in an unselected population [2–4]. Described risk factors are old age, obesity and foot or leg deformities. Further data concerning histology, symptoms and alternative therapy methods are given elsewhere [5, 6].

Our first randomized trial on plantar fasciitis [5] comparing the analgesic effect of a standard dose (1.0Gy two times a week up to a total dose of 6Gy) to that of a very low dose (0.1Gy two times a week up to a total dose of 0.6Gy) resulted in a highly significant superiority of the standard dose. Thus, to our opinion the analgesic effect of low-dose radiotherapy for painful heel spur could be confirmed.

In the last 15 years, in-vitro data have shown that single doses in the range 0.5-0.7Gy might be even more effective than higher ones. Roedel et al. published in 2002 that single doses of 0.3-0,5 Gy lead to a local maximum of apoptosis in macrophages and a reduced E-selectin-presentation on endothelium cells combined with an enhanced expression of TGFß1 [7]. Hildebrand could also show in vitro that depending on modulation of cytokine-stimulated E-selectin-presentation leukocytes could less adhere to endothelium especially with single doses of 0.3-0.6 Gy. This finding also underlines the anti-inflammatory effect of low-dose therapy [8]. Additionally, Gaipl et al. (2009) revealed a maximum of activity-induced cell death in polymorphnuclear cells by the use of single doses of 0.3 Gy in radiotherapy [9]. Further anti-inflammatory effects of low-dose radiotherapy seem to be a reduced CCL-20-chemokine-expression, a reduced adhesion of granulocytes to endothelium cells and an enhanced activity of AP-1 DNA ligation strength [10]. An actual review article stresses the increased expression of the X-linked inhibitor of apoptosis and TGFß1 and the reduced expression of E- and L-selectin, Interleukin-1 and CCL20 through macrophages and polymorphnuclear cells using single doses between 0.5 and 0.7 Gy [11]. Another review article shows additionally high levels of hemioxigenase 1 (HO-1) and heat shock protein 70 (HSP 70) at a maximum using single doses of 0.5 Gy [12].

Thus, we conducted a second clinical prospective randomized multicenter trial in order to compare the analgesic effect of a (to that time) standard single dose of 1Gy to that of a lower dose of 0.5Gy keeping the total dose constant.

## Patients and methods

In this prospective multicenter randomized trial patients were included if meeting the following criteria:

- Clinical evidence of a painful heel spur
- Radiological proof of the spur (plain lateral radiographs of the heel)
- Duration of anamnesis more than six months
- Karnofsky performance status > =70 %
- Age > = 40 years.

### Patients showing

- Previous radiation therapy to the foot
- Previous trauma to the foot
- Rheumatic, arterial or venous diseases, or manifest lymphatic edema of the concerned foot/leg
- Pregnancy or breastfeeding
- Severe psychiatric disorders

were considered ineligible for this trial.

Patients with a long duration of anamnesis and refractory to former treatments could be enrolled. The use of analgesics before and after enrolment was not limited. Patients having undergone surgery or shock wave therapy after randomization were excluded.

All patients gave their written informed consent to radiation therapy and to participate in this trial before enrolment. They were randomly assigned by the statistician (G.S.) to one of the following groups:

- Standard dose group: total dose of 6 Gy applied in single fractions of 1Gy twice a week
- Experimental dose group: total dose of 6Gy applied in single fractions of 0.5 Gy three times a week.

Radiotherapy sessions were performed on Monday/Thursday or Tuesday/Friday (standard dose group) and on Monday/Wednesday/Friday (experimental dose group) to avoid treatment sessions on consecutive days.

Follow-up examinations were scheduled every six weeks either by an examination of the patient in the clinic or by mailing questionnaires to the patients. All patients are intended to receive a follow-up for 48 weeks. The duration of follow-up was chosen on the basis of our retrospective experience that the vast majority of beneficial effects became apparent after less than one year.

### Primary endpoints were

- SF12 sum score (high values = good quality of life) [13]
- Calcaneodynia pain sum score (100 = free of symptoms, 0 = very intense symptoms) [14, 2]
- Visual analogue scale (0 = no pain, 100 = maximum imaginable pain intensity)

Secondary endpoints were the SF12 single scores, Calcaneodynia single scores and the event-free interval.

Radiation therapy was performed using 6 MV- photons of a linear accelerator applying lateral opposing portals. The calcaneus and the plantar aponeurosis were included in the target volume. The dose was prescribed to the ICRU reference point in the center of the calcaneus. The dose was calculated individually according to the clinician’s measurements.

The trial protocol was approved by the Ethics committee of the Saarland Physicians’ Chamber (number 14/07 on 09/05/2012). Furthermore, it has been approved by the expert committee of the DEGRO (German Society for Radiation Oncology). The research carried out here is in compliance with the Declaration of Helsinki in its current version.

Randomization was performed as a block randomization by the statistician. Patients were assigned randomly to one of the therapy arms by equal possibility for randomization for both arms.

According to a previous trial published by Niewald et al. [15, 5] 120 patients are required in each arm and have to be evaluated over 48 weeks (inclusive a drop-out rate of 10 % of the patients) in order to detect a difference of 15 % in VAS and Calcaneodynia-scores with a power of 80 % (error probability of 5 %).

The categorical variables (patients’ demographic and disease data (see Table 1) were compared using the chi-square test and Fisher’s exact test, respectively. Because they were not normally distributed, the pain and quality of life scores in the groups were compared using the Mann–Whitney-*U*-test (see Tables 2 and 3). P-values < =0.05 were considered as statistically significant.

**Table 1:** Comparison of patient data

Item	Standard dose group (*n* = 59)	Experimental dose group (*n* = 58)	p
Mean age (years)			
	56.1	58.1	
SD	9.2	8.7	0.207
			(*t*-Test)
Localization of spur			
Plantar	58 (98 %)	54 (93 %)	
Plantar and dorsal	1 (2 %)	4 (7 %)	0.350
Mean duration of pain (months)	16.5	16.7	0.882
SD	21.2	23.2	
Localization of pain			
Right = left		5 (7 %)	
Right > left	5 (8 %)	2 (3 %)	
Left > right	9 (16 %)	1 (2 %)	
Right only	26 (44 %)	22 (38 %)	
Left only	19 (32 %)	29 (50 %)	0.004
Extension of pain			
None	21 (36 %)	20 (34 %)	
Calf	15 (25 %)	17 (29 %)	
Sole of foot	16 (27 %)	15 (26 %)	
both	7 (12 %)	6 (11 %)	0.979
Start of pain			
Not known	2 (3 %)	1 (2 %)	
Suddenly	23 (39 %)	24 (41 %)	
Insidious	34 (58 %)	33 (57 %)	>0.999
Impact of pain on life quality			
No impact		3 (5 %)	
Leisure	4 (7 %)	7 (12 %)	
Work	3 (5 %)	1 (2 %)	
Leisure and work	52 (88 %)	47 (81 %)	0.165
Effects on daily work			
Able to work	49 (83 %)	52 (90 %)	
Unable to work	7 (12 %)	2 (3 %)	
No occupancy	3 (5 %)	4 (7 %)	0.258
Effects on leisure/sports			
Unlimited	1 (2 %)	3 (5 %)	
Limited	40 (68 %)	37 (64 %)	
Impossible	16 (27 %)	17 (29 %)	
No sports	2 (3 %)	1 (3 %)	0.722
Former therapy using			
Ice/heat	7 (12 %)	3 (5 %)	0.322
Ultrasound	8 (14 %)	15 (26 %)	0.108
Microwaves	1 (2 %)	1 (2 %)	>0.999
Oral medication	36 (61 %)	39 (67 %)	0.564
Injections	19 (32 %)	23 (40 %)	0.444
Heel splints	56 (95 %)	55 (95 %)	>0.999
Other shoe adjustment	4 (7 %)	4 (7 %)	>0.999
Surgery	1 (2 %)	1 (2 %)	>0.999
(Multiple choices possible)			

*SD* standard deviation

**Table 2:** Comparison of pain/quality of life data before radiation therapy

Item	Value	Standard dose group (*n* = 54)	Experimental dose group (*n* = 53)	p
VAS score	Mean	71.8	67.2	
	SD	13.9	13.2	
	Minimum	40	25	
	Maximum	100	90	
	p			0.069
CS sum score	Mean	41.9	47.5	
	SD	10.1	8.7	
	Minimum	14	23	
	Maximum	62	68	
	p			0.002
SF12 somatic, doctor	Mean	38.4	39.5	
	SD	9.9	9.2	
	Minimum	16	18	
	Maximum	56	54	
	p			0.520
SF12, psychic, doctor	Mean	56.7	59.0	
	SD	8.6	8,0	
	Minimum	33	38	
	Maximum	70	74	
	p			0.133
SF12, somatic, patient	Mean	39.2	40.8	
	SD	9.9	8.9	
	Minimum	16	16	
	Maximum	57	54	
	p			0.344
SF12, psychic, patient	Mean	55.5	56.9	
	SD	8.6	8.2	
	Minimum	31	28	
	Maximum	70	71	
	p			0.352

*CS* calcaneodynia score, *VAS* visual analogue scale, *SD* standard deviation

**Table 3:** Comparison of pain/quality of life data 12 weeks after radiation therapy to those before radiation therapy

Item	Value	Standard dose group difference (*n* = 54)	Experimental dose group difference (*n* = 53)	p
VAS (12)-VAS(0)	Mean	−42.3	−44.4	
	SD	27.4	22.8	
	Minimum	−90	−90	
	Maximum	20	0	
	p			0.778
CS(12)-CS(0)	Mean	28.0	28.4	
	S.D.	21.9	20.5	
	Minimum	−16	−22	
	Maximum	68	65	
	p			0.779
SF12, somatic, doctor (12)-(0)	Mean	8.8	10.4	
	SD	11.8	11.2	
	Minimum	−16	−27	
	Maximum	40	34	
	p			0.321
SF12, psychic, doctor (12)-(0)	Mean	−0.6	- 0.3	
	SD	10.9	8.1	
	Minimum	−29	−20	
	Maximum	17	25	
	p			0.662
SF12, somatic, patient (12)-(0)	Mean	8.2	8.5	
	SD	11.2	10.8	
	Minimum	−16	−27	
	Maximum	40	36	
	p			0.559
SF12, psychic, patient (12)-(0)	Mean	0.5	1.2	
	S.D.	10.5	7.9	
	Minimum	−27	−21	
	Maximum	18	17	0.952
	p			

VAS(0), CS(0), SF12(0): values before start of radiation therapy

VAS(12), CS(12), SF12(12): values after 12 weeks’ follow-up

VAS scale: linear scale, 0 = no pain, 100 = maximum imaginable pain, improvement = negative difference, worsening = positive difference

CS scale: linear scale based on criteria like pain, use of aids, problems at work, in daily life, and performing sports, gait: 0 = maximum pain and disability, 100 = complete freedom from symptoms; improvement = positive difference, worsening = negative difference

SF12 scales: complex scales using 12 items on quality of life; high values = favorable quality of life; improvement = positive difference, worsening = negative difference

*SD* standard deviation

The statistical computations were performed using the MEDLOG software package (Fa. Parox, Münster) after observing the patients for 12 weeks and were controlled by the statistician. Further details of this trial protocol have been published elsewhere [5].

## Results

A total of 127 patients were included in this trial. A majority of 112 patients was treated in the University Hospital of Homburg, ten in the University Hospital of Regensburg, and the remaining five in the University Hospital of Mainz.

Nine patients had to be excluded after randomization either due to their refusal to undergo therapy or to radiotherapy with an incorrect dosage (that of the other arm). A further patient was excluded due to a critical lack of data. Finally, 59 patients were assigned to the standard dose group and the remaining 58 to the experimental dose group. Of those, 54 patients in the standard dose group and 53 in the experimental dose group could be followed-up for twelve weeks.

### Comparison of patient groups

The mean age at enrolment was 56.1 years (standard dose group) and 58.1 years (experimental dose group, *p* = 0.207). The mean duration of pain anamnesis was 17 (standard dose group) vs. 16 months (experimental dose group, *p* = 0.882). Concerning localization of spur, extension and start of pain, impact on quality of life, daily work and sports, and former therapy we did not find any significant differences between the groups. A significant imbalance was noticed concerning the localization of pain (*p* = 0.004). Details are summarized in Table 1.

The VAS scores before radiation therapy were distributed equally between the groups (*p* = 0.069) whereas the Calcaneodynia pain score (CS) was found incomparable (*p* = 0.002) with a mean value of 41.9 in the standard dose group and of 47.5 in the experimental dose group indicating a slightly worse pain situation in the standard dose group. There were no statistically significant different values between the groups concerning the SF 12 results (psychic and somatic problems, patient’s and doctor’s evaluation). The detailed data are summarized in Table 2.

### Results after three months’ follow-up

The mean difference of the VAS scores after three months compared to the values before radiation therapy was 42.3 in the standard dose group and 44.4 in the experimental dose group (*p* = 0.778). A similar result was found when evaluating the CS score (*p* = 0.779). Consequently, no statistically significant difference could be found between the groups.

The results concerning quality of life fitted well to those concerning pain. No statistically significant differences could be found (somatic scale, doctor’s judgement: *p* = 0.321; psychic scale, doctor’s judgement: *p* = 0.662; somatic scale, patient’s judgement: *p* = 0.559; psychic scale, patient’s judgement: *p* = 0.952).

No relevant side effects were recorded.

## Discussion

The aim of this multicenter randomized study was to compare the analgesic effect of standard single doses compared to lower ones keeping the total dose constant. At the time this trial was planned, the standard dose was 1.0Gy twice a week up to a total dose of 6Gy. Compared to this dosage we did not find any difference in our experimental arm (12 times 0.5Gy three times a week). In the meantime, a new standard of single doses or 0.5Gy twice a week up to a total dose of 3.0Gy has been established according to the results published by Heyd et al. [16] and Ott et al. [17]. Compared to this standard we did not see any benefit after escalation of the dose to 6Gy. In summary, we could not find any difference resulting in the assumption that the in-vivo results could not be translated into clinical pain relief.

The authors are well aware of the limitations of this trial. Because of the results of the interim analysis shown here without a reasonable probability to see a significant difference between the groups by recruiting further patients the study was closed prematurely which may limit its statistical power. A further reason for the early closure were the results published by Ott et al. [17] showing that the same grade and duration of pain relief can be achieved applying a total dose of 3 Gy in single fractions of 0.5 Gy twice a week compared to the known standard dose of 6 Gy in single fractions of 1.0 Gy twice a week at a high level of evidence. In the meantime, the standard total dosage has been lowered to 3 Gy in many institutions in Germany according to the ALARA principle (*keep the dose* as low as reasonably achievable). Thus, continuation of the trial could have lead to an ethical problem.

Furthermore, the trial was conducted in a randomized manner but not blinded to the patient or the physician. Using a linear accelerator, it seemed nearly impossible and unethical to perform a blinded radiotherapy. Finally, the results may have been confounded by the fact that concomitant therapy with oral analgesics was not limited. Otherwise, it may be assumed that limitation of concomitant medication would have caused an unwanted selection bias.

Numerous retrospective trials have shown that low-dose radiotherapy for painful heel spur has a good analgesic effect, pain relief has been noticed in 65 – 90 % of the patients (literature table in [18]). However, a certain placebo effect is still under discussion [19]. Goldie et al. examined this effect in 399 patients. They found a response in 60 % of the patients whether irradiated or not; these results made the effect of radiotherapy questionable [20]. The trial, however, has been criticized because of missing clearly defined endpoints: furthermore the therapy was started in an acute stage of the diseases and the authors did not wait for spontaneous pain remissions. In the meantime, several more modern trials have shown the analgesic effect of radiotherapy. Seegenschmiedt et al. [3] performed a randomized trial treating 141 patients (170 heels) for painful heel spur using orthovoltage, comparing three radiotherapy schedules: 1 Gy/fraction up to 12 Gy, 0.3 Gy/fraction up to 3 Gy and 0.5 Gy/fraction up to 5 Gy. The overall complete pain relief was reported in 67–72 % of the patients. The best results were seen after a total dose of 5 Gy. These results were confirmed by Schäfer et al. using a telecobalt machine, they achieved a complete pain relief in 58 % [33]. Heyd et al. used 6 MV photon beams of a linear accelerator, they noticed a frequency of pain relief of 69 % [4]. The same author group published a prospective randomized trial recently [16] comparing the effect of a total dose of 3 Gy (single fraction 0.5 Gy twice a week) to that of a total dose of 6 Gy (single fraction 1 Gy twice a week). Radiotherapy was reported very efficient, however a dependency on dose could not be noticed. Mücke et al. looked for prognostic factors for pain relief in a multicenter trial [18]. They found an overall response in 60.9 %. Significant favourable prognostic factors for pain relief were a patient's age over 58 years, the use of megavoltage techniques and the number of therapy series required. Niewald et al. showed in a recent prospective study that a total dose of 6 Gy with single doses of 1 Gy twice a week is much more effective than a total dose of 0.6 Gy with single doses of 0.1 Gy twice a week concerning analgesic effect and showed that a total doses of 6 Gy makes this effect durable for at least 1 year [5]. The important results published by Ott et al. [17] have been mentioned earlier.

## Conclusions

Radiation therapy yields important pain relief in patients with painful heel spur (plantar fasciitis). The very encouraging laboratory results showing an enhanced effect of single doses in the range of 0.5-0.7 Gy compared to higher ones could not be translated into clinical practice. Until now we found no plausible explanation for this result. Further trials based on a total dose of 3 Gy will be necessary to explore the best dose schedule. This trial will be continued until all patients have reached a follow-up duration of 48 weeks. Only these long-term results will allow a final decision.

## Abbreviations

AP-1: Activator protein-1
CCL: Chemocine ligand
cm: Centimeter(s)
CS: Calcaneodynia score
DEGRO: German Society for Radiation Oncology
GCGBD: German Cooperative Group for Radiotherapy for Benign Diseases
Gy: Gray
iNOS: Inducible nitric oxide synthetase
kV: Kilovolt
MR: Magnetic resonance
MV: Megavolt
NO: Nitric oxide
pH: Pondus hydrogenii
SD: Standard deviation
SF-12: Short Form-12
TGFß1: Transforming growth factor ß1
VAS: Visual analogue scale
